# Trend and spatial clustering of medical education in Brazil: an ecological study of time series from 2010 to 2021

**DOI:** 10.1186/s12913-023-09795-9

**Published:** 2023-08-22

**Authors:** Rafael Alves Guimarães, Ana Luísa Guedes de França e Silva, Marizélia Ribeiro de Souza, Adriana Moura Guimarães, Marcos Eduardo de Souza Lauro, Alessandra Vitorino Naghettini, Heliny Carneiro Cunha Neves, Fernanda Paula Arantes Manso, Cândido Vieira Borges Júnior, Alessandra Rodrigues Moreira de Castro, Victor Gonçalves Bento, Pablo Leonardo Mendes da Cruz Lima

**Affiliations:** 1 Nursing School, Federal University of Goiás, Goiânia, GO Brazil; 2 Institute of Tropical Pathology and Public Health, Federal University of Goiás, Goiânia, GO Brazil; 3 Center for Innovation in Education and Health Work Management, Federal University of Goiás, Goiânia, GO Brazil; 4 Medical School, Federal University of Goiás, Goiânia, GO Brazil; 5 Administration, Accounting , Economic Sciences School, Federal University of Goiás, Goiânia, GO Brazil; 6 Federal District, Ministry of Health, Brasília, Brazil; 7 Computing Resource Center, Federal University of Goiás, Goiânia, GO Brazil

**Keywords:** Medical education, Trend, Spatial clusters

## Abstract

**Context:**

Studies that analyze the temporal trend and spatial clustering of medical education indicators are scarce, especially in developing countries such as Brazil. This analysis is essential to subsidize more equitable policies for the medical workforce in the states and regions of Brazil. Thus, this study aimed to analyze the temporal trend and identify spatial clusters of medical education indicators in Brazil disaggregated by public and private education, states, and regions.

**Methods:**

A time-series ecological study was conducted using data from the Higher Education Census of the Ministry of Education from 2010 to 2021. The study analyzed vacancy density indicators of active and former students/100,000 population, disaggregated by public and private education, 27 states, and 5 regions in Brazil. Prais-Winsten regression was used for trend analyses of indicators. Hot Spot Analysis (Getis-Ord Gi*) was used to identify spatial clusters of indicators.

**Results:**

The number of medical schools increased by 102.2% between 2010 and 2021. A total of 366 medical schools offered 54,870 vacancies at the end of 2021. Vacancy density and active and former students increased significantly in the period, but this increase was greater in private institutions. Most states and regions showed an increasing trend in the indicators, with higher increase percentages in private than in public schools. Hot spot spaces changed over time, concentrated in the southeast, center-west, and north at the end of 2021. Medical education remains uneven in Brazil, with a low provision in regions with low socioeconomic development, academic structure, and health services, represented by regions in the north and northeast.

**Conclusions:**

There is a growing trend in medical education indicators in Brazil, especially in the private sector. Spatial clusters were found predominantly in the southeast, center-west, and north. These results indicate the need for more equitable medical education planning between the regions.

## Background 

Medical education has undergone a significant vacancy expansion process in Brazil, especially in the private education sector. This expansion is influenced by multiple factors, especially political decisions and scenarios, the current economic model, and public health and education policies [1]. These factors have defined the trend, expansion, and geographic distribution of medical education in the country [1, 2]. This phenomenon of medical education expansion has followed a global trend observed in emerging economies, which have increased the number of medical schools due to workforce shortage and the increased demand for health care by the population caused, above all, by aging and epidemiological and nutritional transition [3–5].

The number of medical courses and vacancies began to grow in Brazil in the 1960s, with the creation of 35 medical schools. This expansion intensified in the twenty-first century. At the end of 2020, there were 328 active medical courses, totaling 35,480 authorized vacancies for admission [6]. This recent growth process is related to Law number 12,871/2013, which created the More Doctors Program (in Portuguese, *Programa Mais Médicos* [PMM]) [7]. On December 18, 2019, Law number 13,958 renamed the program to More Doctors for the Brazil Program (in Portuguese, *Programa Mais Médicos pelo Brasil*) [8], now called Ministry of Health's Doctors Provision Program—More Doctors for Brazil Project (in Portuguese—*Programa de Provisão de Médicos do Ministério da Saúde—Projeto Mais Médicos para o Brasil*). The main objective of the PMM is to assure health services access for the population, especially in more socioeconomically vulnerable areas with poor access to health services [7, 8]. As a result of medical workforce concentration in capitals and metropolitan regions and the structural deficiency in socioeconomically more vulnerable regions, the PMM significantly expanded the number of vacancies in medical courses and medical residency programs [9, 10]. Medicine courses are maintained, predominantly, by private institutions and medical residency programs by public funds [9, 10]. The PMM was also responsible for some changes in medical education, including its orientation towards primary health care. Thus, the program proposes a theoretical-practical curriculum focused on primary health care. This aimed at training physicians referred to primary care. In addition, it allowed, even in its first version of the Program in 2013, that professionals from other countries began to provide care in Brazil to meet the supply of doctors in needy areas, predominantly Cubans who entered through an agreement between Cuba and Brazil, organized by the Pan American Health Organization (PAHO) [9, 11, 12]. This expansion was also related to the higher education growth in Brazil, which multiplied the number of schools and vacancies in undergraduate health courses in general [2].

Other initiatives to attract and retain physicians in more vulnerable regions also contributed to this medical education expansion in the country. Some programs are highlighted, including the Unified Health System Interior Growth Program (in Portuguese, *Programa de Interiorização do Sistema Único de Saúde [*PISUS]) (1993) [13], the Health Work Growth in Interior Cities Program (in Portuguese, *Programa de Interiorização do Trabalho em Saúde* [PITS]) (2001) [14], the Support Program to Restructure and Expand Federal Universities (in Portuguese, *Programa de Apoio à Reestruturação e Expansão das Universidades Federais* [REUNI]) (2007) [15], the Program for the Valorization of Primary Care Professionals (in Portuguese, *Programa de Valorização dos Profissionais da Atenção Básica* [PROVAB]) (2011) [16], and the National Policy for the Expansion of Medical Schools of Federal Higher Education Institutions (2013) [17], in addition to policies such as an increase of government incentives in education for the public funding of scholarships and tuition fees in private schools [6]. All these programs and policies contributed to expanding medical education in the country.

However, despite programs and policies to expand medical education, some studies show a shortage of these professionals, especially in less socioeconomically developed regions and in PHC [18]. A previous study showed deficits and inequalities in the ratio of physicians to the population between Brazilian regions in 2020, especially in the north and northeast, which have a lower level of socioeconomic development, academic infrastructure, and healthcare network [10]. Regions in the south, southeast, and center-west, which have higher development levels, had ratios of physicians/1,000 population of 3.15, 2.68, and 2.74, respectively. However, in the northeast and north, these ratios were 1.30 and 1.69 physicians/1,000 population, respectively [10]. This scenario indicates that inequalities persist even with expansion programs and policies to attract and retain physicians in less developed regions. Following the global scenario, these data suggest that Brazil has issues related to actions to influence the homogeneous distribution, establishment, supply, and training of physicians [11].

Previous studies analyzed the distribution of indicators such as vacancy density and former medical students in Brazil, including differences between the public and private sectors. For example, a study reported 241 medical schools in 2014, totaling 20,340 vacancies, showing that private HEIs (in Portuguese, *Instituições de Ensino Superior* [IES]) were responsible for more than half of the medical student enrollments in the country (54.0%). Most vacancies and enrollments were concentrated in the southeast region, the most developed in the country [11]. A study conducted in 2020 showed that most of the 35,480 vacancies were in private institution courses (74.1%), an increase of almost 20.0% compared to 2014. It also showed that most vacancies were provided in regions with high and very high human development indices (HDI), being concentrated in the southeast region, which has approximately the total number of vacancies, a result similar to that found in the 2020 medical demography study [6, 10]. Another observational study showed that 19,519 new vacancies were created in medical courses in Brazil from 2010 to 2018, an increase of 120.2%. Simultaneously, the medical workforce increased in the labor market and the Unified Health System (in Portuguese, *Sistema Único de Saúde* [SUS]). The study also showed that some policies, such as the PMM and the expansion of federal medical schools, reduced medical education access inequalities and supplied physicians to cities with smaller populations, lower Gross Domestic Product (GDP) per capita, and a lower ratio of physicians per population [19].

Despite previous studies [6, 10, 11, 19], there is a gap in the literature about the temporal trend of indicators, including vacancy and former students densities, especially in a recent period (2021). Trend analyses disaggregated by public and private education and states and regions are limited in Brazil. Although previous studies showed a concentration of courses and vacancies in the southeast and south, no studies found in the literature analyzed spatial clusters of indicators, including those disaggregated by public and private institutions [6, 10, 11]. These assessments, added to the present study, are essential for analyzing concentrations and deficits in physician provision and training in Brazil. The analyses proposed in this study can help direct policies and programs to distribute, supply, and train physicians in Brazil, allowing the health workforce to be planned according to the needs and characteristics of each region. The present study also adds data that can help strengthen the World Health Organization’s (WHO) global strategy on human resources for health, Workforce 2030 [20], by identifying the states and regions with the lowest medical education supply in Brazil. It also helps systems move toward universal health coverage to achieve several Sustainable Development Goals (SDGs) [21]. Thus, this study aimed to analyze the temporal trend and identify spatial clusters of medical education indicators in Brazil disaggregated by public and private education, states, and regions.

## Methods

An ecological time-series study analyzed trends in vacancy and active and former students densities in medical courses disaggregated by public and private teaching, states, and regions. We also analyzed the spatial clusters of these indicators.

**Fig. 1:**
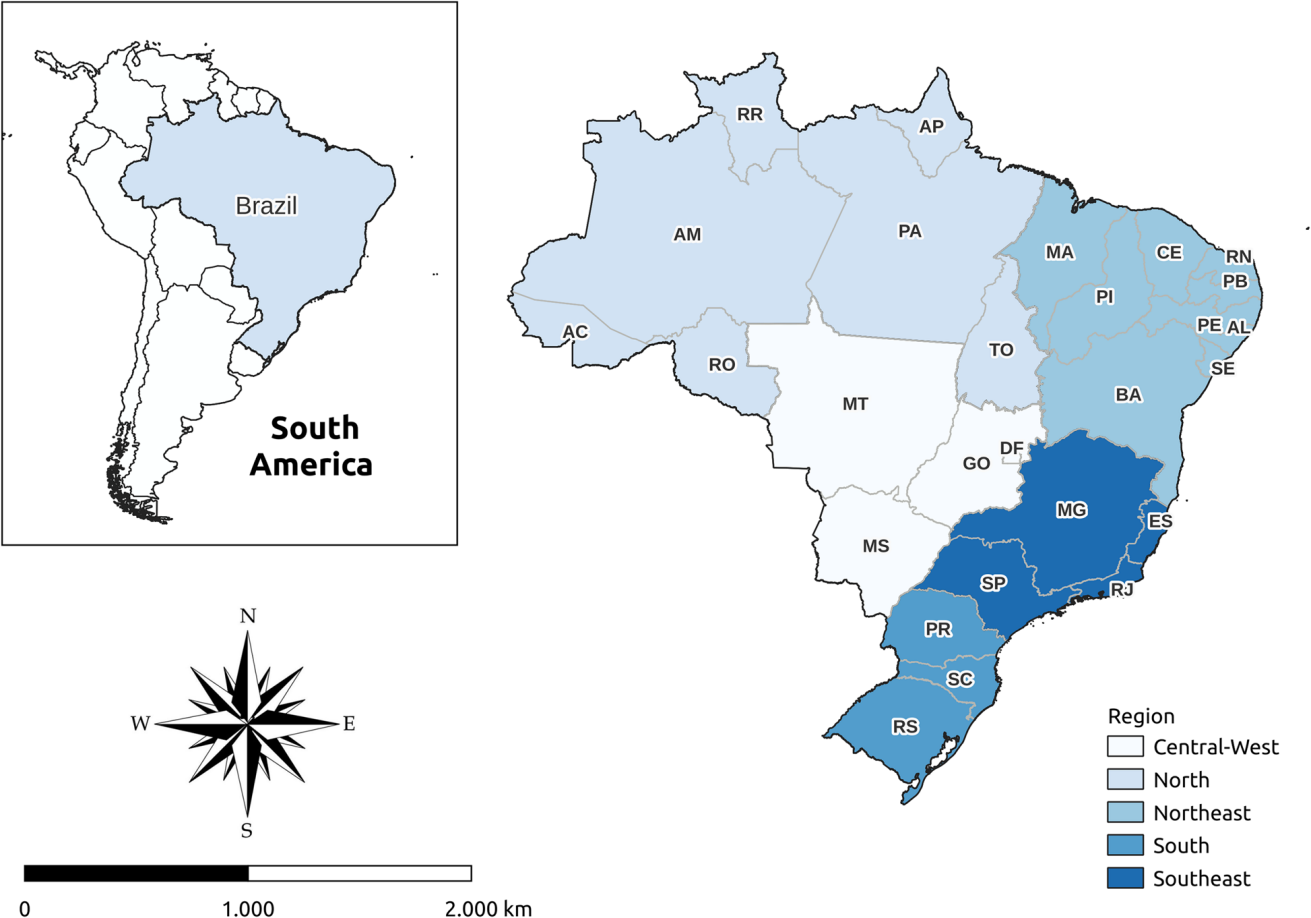
Study scenarios. Abbreviations: AC = Acre; AL = Alagoas; AP = Amapá; AM = Amazonas; BA = Bahia; CE = Ceará; DF = Distrito Federal; ES = Espírito Santo; GO = Goiás; MA = Maranhão; MT = Mato Grosso; MS = Mato Grosso do Sul; MG = Minas Gerais; PA = Pará; PB = Paraíba; PE = Pernambuco; PI = Piauí; PR = Paraná; RJ = Rio de Janeiro; RN = Rio Grande do Norte; RO = Rondônia; RR = Roraima; RS = Rio Grande do Sul; SC = Santa Catarina; SE = Sergipe; SP = São Paulo; TO = Tocantins

The study was conducted using data from 2010–2021. Data from all Brazilian states were included. The country had an estimated population of 221 million people in 2021, distributed in 5,570 cities. Of these cities, 67.7% have a low population (less than 20,000 people). Cities are grouped into 26 states and the Federal District, which are grouped into 5 major regions, that is, center-west, northeast, north, southeast, and south (Fig. 1), which have different demographic and socioeconomic characteristics and health service structures, among other aspects [22]. Regions in the northeast and north have the lowest GDP per capita, physicians per population ratio, and the number of health institutions, while the southeast has the highest level of socioeconomic development and health service infrastructure. Brazil has a territorial extension of 8,510,345.540 km[2] and a population density of 26.0 people/km[2]. The GDP per capita is BRL 35,935.74, the illiteracy rate in 15-year-old or older people is 6.6%, and the HDI is 0.754, ranking 87 in the development ranking of 191 countries according to the latest available data [22, 23].

We used microdata from the Higher Education Census by the Anísio Teixeira National Institute of Educational Studies and Research (in Portuguese, *Instituto Nacional de Estudos e Pesquisas Educacionais Anísio Teixeira* [INEP]) of the Brazilian Ministry of Education as the main data source [24, 25].

The Higher Education Census is an annual survey that uses higher education institutions (HEIs), courses, students, and professors as sources of information. The population includes HEIs registered in the Ministry of Education computerized system with at least one active course with at least one student in the year of the Higher Education Census. It encompasses several programs, including the medical course. The census is mandatory for all public and private HEIs. Only institutions with no students linked to the HEI in the reference year are not obliged to answer the census. The legal representative of the HEI is responsible for appointing the institutional researcher, the person who will provide information to the Ministry of Education. Data collection has undergone methodological changes in recent years, with data now being collected through an online system. The institutional researcher inserts multiple data, such as HEIs, courses, professors, and students, among others. All people completing the census are duly trained on the procedures and fields to be filled out. Other methodological details can be consulted in a previous publication [25].

The following variables were extracted from the microdata: (i) the number of medical schools, (ii) the total number of vacancies in medical courses, (iii) the number of students enrolled in medical courses, (iv) the number of former students from medical courses, (v) types of institution (public or private), and (vii) the cities, states, and regions where medical schools are located. We used the criteria of the 1996 Law of Educational Guidelines and Bases (in Portuguese, *Lei de Diretrizes e Bases da Educação* [LDB]) to define public and private institutions [26], with public institutions defined as the ones created, incorporated, maintained, and managed by public authorities, irrespective of whether federal, state, or municipal. Public institutions can be directly managed by the government or indirectly by foundations or autonomous public entities [11]. Private institutions are those maintained and managed by individuals or legal entities governed by private law [26]. As defined by Scheffer et al. [11], the terms “medical school” and “medical course” refer to autonomous structures that provide undergraduate medical education, and such terms were used in this study.

Resident population data were extracted from the 2010 demographic census and 2011–2021 intercensal projections by the Brazilian Institute of Geography and Statistics (in Portuguese, *Instituto Brasileiro de Geografia e Estatística* [IBGE]) [27].

From the extracted variables, the following indicators were analyzed:

$$\left(\mathrm{i}\right)\text{Vacancy density}=\frac{\text{Total number of vacancies}}{\text{Total resident population}}\text{ x 100,000}$$

$$\left(\mathrm{ii}\right)\text{ Enrolled student density }=\frac{\text{Total number of enrolled students}}{\text{Total resident population}}\text{ x 100,000}$$

$$\left(\mathrm{iii}\right)\text{ Former students density }=\frac{\text{Total number of former students}}{\text{Total resident population}}\text{ x 100,000}$$

These indicators were disaggregated by public or private institutions, states, regions, and Brazil.

Trends analysis were analyzed using R version 4.3.1, with interface RStudio [28]. The analysis units of the trend study consisted of the time-series years (2010–2021). We used the Prais-Winsten linear regression model with robust variance adjusted for Durbin-Watson autocorrelation [29] to assess the trend of indicators disaggregated by public and private institutions, states, and regions. Three indicators were included as dependent variables (Y): (i) vacancy density/100,000 population, (ii) density of enrolled students/100,000 population, and (iii) density of former students/100,000 population. A log base 10 transformation was performed before inclusion in the regression models to reduce the heterogeneity of the residual variance, thus, contributing to the temporal trend determination [29]. The year was included as an independent variable (X). The Prais-Winsten regression equation is defined by ${\mathrm{Log}(\mathrm{Y}}_{\mathrm{t}})={\upbeta }_{0}+{\upbeta }_{1}+{\mathrm{e}}_{\mathrm{t}}$, being ${\mathrm{Log}(\mathrm{Y}}_{\mathrm{t}})$ the dependent variables, ${\upbeta }_{0}$ the intercept or regression constant, ${\upbeta }_{1}$ the line slope, and ${\mathrm{e}}_{\mathrm{t}}$ the random error; “t” estimates the times of the dataset {t1, …, t12} [29], in case t1 = 2010 and t12 = 2021.

Regression results were used to calculate the annual percentage variation (APV) and its 95% confidence intervals (95% CI). The APV was calculated using the following formula:

$$\mathrm{VPA}={{(1+10)}^{{\upbeta }_{1}}*100},$$

${\upbeta }_{1}$ being the line slope obtained in the regression equation. The APV 95% CI was calculated by the formula:

$$\mathrm{IC}95\mathrm{\%}={(1+10}^{{(\upbeta }_{1}\,\pm\,{t*\mathrm{EP})}})*100,$$

${\upbeta }_{1}$ being the line slope, t is the value in which the Student t distribution has 11 degrees of freedom at a two-tailed 95% CI, and SE is the standard error.

Trends were classified as increasing when the APV was positive, and the *p*-value was significant, or decreasing when the APV was negative, and the *p*-value was significant or stationary, with positive or negative APV, and the *p*-value was not significant. A significance level of 0.05% was adopted (*p*-value < 0.05) from the statistic t [29].

Finally, a spatial analysis of the medical teaching indicators was performed. The unit of analysis was cities in Brazil (*n* = 5,570). Only the series extremes (2010 and 2021) were considered in the analysis. This approach shows the evolution of spatial clusters at the beginning and end of the analytical period. Hot Spot Analysis (Getis-Ord Gi*) [30] was used to identify spatial clusters of indicators. This analysis identifies two types of clusters: Hot Spots as areas of high indicator magnitude and Cold Spots as areas of low magnitude. Contiguity edges were used to conceptualize spatial relationships. The z-score was used to identify significant hot/cold spots. The z-score classified cities as hot or cold areas, with a significance level of 90% (*p*-value < 0.10), 95% (*p*-value < 0.05), or 99% (*p*-value < 0.01) [30, 31]. Details of the Hot Spot Analysis methodology were previously published [30]. Geospatial Hot Spot Analysis (Getis-Ord Gi*) was performed using ArcGIS 10.3 [32].

The study project was approved by the Research Ethics Committee of the Federal University of Goiás, 4,675,978/ 2021. The data did not identify individuals or personal data; thus, written consent was waived.

## Results

Between 2010–2021, the number of medical schools increased from 181 to 366 (Δ: 102.2%). In 2010, medical schools provided a total of 16,583 vacancies, which increased to 54,870 vacancies in 2021 (Δ: 230.88%). Vacancy density/100,000 population ranged between 7.60–22.78 during this period (Δ: 199.74%). The density of active students increased from 41.65–82.53 students/100,000 population (Δ: 98.15%). The density of former students increased from 5.94–10.57 students/100,000 population (Δ: 77.95%) (Table 1).

**Table 1:** Number of courses, vacancies, vacancy density (per 100,000 population), number of former students, and density of former students (per 100,000 population) by state and major regions, Brazil, 2010 and 2021

Regions/States	2010							2021						
	***n***	**Vacancies**	DV*	**Students**	DA*	**Former students**	DF*	***n***	**Vacancies**	DV*	**Students**	DA*	**Former students**	DF*
**North**	19	1457	3.03	8445	17.57	1019	2.12	35	4587	8.81	15,729	30.22	1698	3.26
Acre	1	40	5.45	203	27.67	80	10.91	3	446	49.18	996	109.83	80	13.23
Amapá	1	30	4.48	29	4.33	0	0	1	60	6.84	343	39.08	61	6.95
Amazonas	3	332	9.53	1933	55.48	225	6.46	5	663	15.53	2952	69.13	255	5.97
Pará	4	392	5.17	1961	25.87	379	5	10	1413	16.1	4762	54.25	476	5.42
Rondônia	4	230	14.72	1467	93.89	123	7.87	6	758	41.76	2359	129.95	232	12.78
Roraima	1	33	7.33	172	38.18	0	0	2	113	17.31	506	77.52	83	12.72
Tocantins	5	400	28.91	2680	193.72	212	15.32	8	1134	70.55	3811	237.1	471	29.3
**Northeast**	38	3364	7.00	18,133	37.74	2062	4.29	94	14,101	26.04	46,253	88.86	5254	10.09
Alagoas	2	130	4.17	695	22.27	158	5.06	5	561	16.67	2730	81.12	382	11.35
Bahia	7	586	4.18	3304	23.57	279	1.99	28	5034	33.59	13,048	87.07	1081	7.21
Ceará	7	652	7.71	3183	37.66	432	5.11	11	1394	15.09	5791	62.67	657	7.11
Maranhão	3	230	3.5	1219	18.54	155	2.36	9	826	11.55	3189	44.58	262	3.66
Paraíba	6	530	14.07	3106	82.46	337	8.95	9	1623	39.98	6499	160.08	848	20.89
Pernambuco	4	415	4.72	2662	30.26	370	4.21	14	2171	22.44	8046	83.16	895	9.25
Piauí	4	310	9.94	1731	55.51	196	6.29	8	1382	42.02	3849	117.02	558	16.96
Rio Grande do Norte	3	246	7.77	1185	37.4	92	2.9	6	678	19.04	2785	78.21	404	11.35
Sergipe	2	150	7.25	604	29.21	76	3.68	4	432	18.47	1863	79.67	214	9.15
**Center-West**	12	1002	7.13	5152	36.65	791	5.63	35	5059	30.28	16,639	99.59	2186	13.08
Federal District	4	314	12.22	1791	69.68	307	11.94	6	762	24.63	3312	107.03	429	13.86
Goiás	3	290	4.83	1238	20.62	112	1.87	16	2916	40.46	8555	118.71	958	13.29
Mato Grosso	2	208	6.85	1202	39.6	206	6.79	7	677	18.98	2510	70.36	477	13.37
Mato Grosso do Sul	3	190	7.76	921	37.61	166	6.78	6	704	24.8	2262	79.67	322	11.34
**Southeast**	81	8489	10.56	46,280	57.59	7140	8.88	143	23,663	26.40	91,683	102.29	11,352	12.66
Espírito Santo	5	500	14.22	2551	72.58	308	8.76	6	980	23.85	4118	100.23	516	12.56
Minas Gerais	28	2640	13.47	14,366	73.31	1834	9.36	47	6271	29.29	27,925	130.42	3209	14.99
Rio de Janeiro	18	223	15.15	14,205	88.84	2277	14.24	22	5067	29.02	17,948	102.78	2482	14.21
São Paulo	30	2926	7.09	15,158	36.74	2721	6.59	68	11345	24.32	41,692	89.37	5145	11.03
**South**	31	2271	8.29	12,764	46.60	1937	7.07	59	8008	26.34	28,846	93.69	3274	10.77
Paraná	10	743	7.11	4006	38.36	617	5.91	22	3777	32.57	11,886	102.49	1297	11.18
Rio Grande do Sul	11	969	9.06	5361	50.13	877	8.2	20	2182	19.03	9459	82.49	1226	10.69
Santa Catarina	10	559	8.95	3397	54.37	433	7.09	17	2049	27.92	7141	97.31	751	10.23
**Brazil**	181	16,583	7.60	90,774	41.65	12,949	5.94	366	54,870	22.78	198,790	82.53	23,764	9.87

*DA* Density of active students, *DF* Density of former students, *DV* Density of vacancies

^*^Per 100,000 population

Between 2010 and 2021, the number of medical schools increased from 75–134 (Δ: 78.76%) (Table 2), while private schools increased from 98 to 218 (122.45%) (Table 3). Public schools increased vacancies during the period from 6,642 to 12,033 (81.16%) (Table 2), while private schools increased the same from 9,941 to 42,837 (330.91%) (Table 3). There was a higher percentage of variation between these two years for vacancy density for private schools (Δ: 289.91%, 4.56 to 17.78/100,000 population) (Table 3) compared to public schools (Δ: 63.93%, 3.05 to 5.00/100,000 population) (Table 2). Private schools showed a greater increase in the density of active students (Δ: 130.00%, 26.00 to 59.80 students/100,000 population) (Table 3) compared to public schools (Δ: 45.24%, 15.65 to 22.73 students/100,000 population) (Table 2). Private schools also showed a greater increase in the density of former students (Δ: 109.40%, 3.19 to 6.68/100,000 population) (Table 3) compared to public schools (Δ: 16.00%, 2.75 to 3.19/100,000 population) (Table 2).

**Table 2:** Number of courses, vacancies, vacancy density (per 100,000 population), number of former students, and density of former students (per 100,000 population) in public institutions by state and major regions, Brazil, 2010 and 2021

Regions/States	2010							2021						
	***n***	**Vacancies**	DV*	**Students**	DA*	**Former students**	DF*	***n***	**Vacancies**	DV*	**Students**	DA*	**Former students**	DF*
**North**	11	867	1.80	4653	9.68	720	1.50	18	1354	2.60	6280	12.06	911	1.75
Acre	1	40	5.45	203	27.67	80	10.91	1	90	9.92	396	43.67	59	6.51
Amapá	1	30	4.48	29	4.33	0	0	1	60	6.84	343	39.08	61	6.95
Amazonas	2	232	6.66	1411	40.5	173	4.97	3	256	6	1481	34.68	183	4.29
Pará	3	292	3.85	1562	20.6	379	5	5	376	4.28	2024	23.06	290	3.3
Rondônia	1	40	2.56	238	15.23	42	2.69	1	40	2.2	156	8.59	35	1.93
Roraima	1	33	7.33	172	38.18	0	0	2	113	17.31	506	77.52	83	12.72
Tocantins	2	200	14.46	1038	75.03	46	3.33	5	419	26.07	1374	85.48	200	12.44
**Northeast**	24	1782	3.71	8739	18.19	1444	3.00	42	2658	5.10	13,175	25.31	1726	3.32
Alagoas	2	130	4.17	695	22.27	158	5.06	3	216	6.42	995	29.57	110	3.27
Bahia	5	286	2.04	1553	11.08	239	1.71	11	478	3.19	3330	22.22	324	2.16
Ceará	4	320	3.79	1515	32.53	316	3.74	4	360	3.9	1772	29.89	223	2.41
Maranhão	2	130	1.98	669	10.18	79	1.2	5	363	5.07	1499	20.96	197	2.75
Paraíba	3	280	7.43	1269	33.69	183	4.86	3	285	7.02	1230	30.3	152	3.74
Pernambuco	3	295	3.35	2039	23.18	370	4.21	6	521	5.39	2733	28.25	350	3.62
Piauí	2	130	4.17	596	19.11	118	3.78	4	240	7.3	1065	32.38	172	5.23
Rio Grande do Norte	2	126	3.98	621	19.6	92	2.9	4	263	7.39	1229	34.51	241	6.77
Sergipe	1	100	4.84	552	26.69	76	3.68	2	168	7.18	825	35.28	155	6.63
**Center-West**	6	454	3.23	2236	15.90	404	2.87	21	2538	15.19	8398	50.27	1074	6.43
Federal District	2	154	5.99	836	17.92	153	5.95	2	180	5.82	925	19.18	165	5.33
Goiás	1	110	1.83	559	9.31	112	1.87	11	1804	25.03	5039	69.92	469	6.51
Mato Grosso	1	80	2.28	285	9.39	37	1.22	4	262	1.95	1087	30.47	218	6.11
Mato Grosso do Sul	2	110	2.64	556	22.7	102	4.16	4	292	7.34	1347	47.44	222	7.82
**Southeast**	24	2448	3.05	12,735	15.85	2463	3.06	36	3887	4.34	18,942	21.13	2868	3.20
Espírito Santo	1	80	4.49	423	12.03	95	2.7	1	80	10.28	424	10.32	88	2.14
Minas Gerais	8	888	4.53	4471	22.81	687	3.51	15	1568	7.32	7666	35.8	1026	4.79
Rio de Janeiro	5	660	4.13	3349	20.94	572	3.58	5	717	4.11	3669	21.01	545	3.12
São Paulo	10	820	1.99	4492	10.89	1109	2.69	15	1522	3.26	7183	15.4	1209	2.59
**South**	10	1091	3.986	5745	20.98	958	3.50	17	1596	5.25	7955	26.17	1096	3.60
Paraná	5	376	3.6	1903	18.22	317	3.04	9	615	5.3	3175	27.38	352	3.04
Rio Grande do Sul	5	507	4.74	2729	25.52	474	4.43	7	695	6.06	3375	29.43	526	4.59
Santa Catarina	3	208	3.33	1113	17.81	167	2.67	4	286	3.9	1405	19.15	218	2.97
**Brazil**	75	6642	3.05	34,108	15.65	5989	2.75	134	12,033	5.00	54,750	22.73	7675	3.19

*DA* Density of active students, *DF* Density of former students, *DV* Density of vacancies

^*^Per 100,000 population

**Table 3:** Number of courses, vacancies, vacancy density (per 100,000 population), number of former students, and density of former students (per 100,000 population) in private institutions by state and major regions, Brazil, 2010 and 2021

Regions/States	2010							2021						
	***n***	**Vacancies**	DV*	**Students**	DA*	**Former students**	DF*	***n***	**Vacancies**	DV*	**Students**	DA*	**Former students**	DF*
**North**	8	590	1.23	3792	7.89	299	0.62	17	3233	6.21	9449	18.15	787	1.51
Acre	0	0	0	0	0	0	0	2	356	39.26	600	66.16	61	6.73
Amapá	0	0	0	0	0	0	0	0	0	0	0	0	0	0
Amazonas	1	100	2.87	522	14.98	52	1.49	2	407	9.53	1471	34.45	72	1.69
Pará	1	100	1.32	399	5.26	0	0	5	1037	11.81	2738	31.19	186	2.12
Rondônia	3	190	12.16	1229	78.66	81	5.18	5	718	39.55	2203	121.36	197	10.85
Roraima	0	0	0	0	0	0	0	0	0	0	0	0	0	0
Tocantins	3	200	14.46	1642	118.69	166	12	3	715	44.48	2437	151.61	271	16.86
**Northeast**	14	1582	3.29	9394	19.55	618	1.29	52	10,895	20.93	33,078	63.55	3528	6.78
Alagoas	0	0	0	0	0	0	0	2	345	10.25	1735	51.55	272	8.08
Bahia	2	300	2.14	1751	12.49	40	0.29	17	4556	30.4	9718	64.85	757	5.05
Ceará	3	332	3.93	1668	19.73	116	1.37	7	1034	11.19	4019	43.49	434	4.7
Maranhão	1	100	1.52	550	8.37	76	1.16	4	463	6.47	1690	23.63	65	0.91
Paraíba	3	250	6.64	1837	48.77	154	4.09	6	1338	32.96	5269	129.78	696	17.14
Pernambuco	1	120	1.36	623	7.08	0	0	8	1650	17.05	5313	54.92	545	5.63
Piauí	2	180	5.77	1135	36.4	78	2.5	4	1142	34.72	2784	84.64	386	11.74
Rio Grande do Norte	1	120	3.79	564	17.8	0	0	2	415	11.65	1556	43.7	163	4.58
Sergipe	1	50	2.42	52	2.51	0	0	2	264	11.29	1038	44.39	59	2.52
**Center-West**	6	548	3.90	2916	20.74	387	2.75	14	2521	15.09	8241	49.33	1112	6.65
Federal District	2	160	6.23	955	37.16	154	5.99	4	582	18.81	2387	77.14	264	8.53
Goiás	2	180	3	679	11.31	0	0	5	1112	15.43	3516	48.79	489	6.79
Mato Grosso	1	128	4.22	917	30.21	169	5.57	3	415	11.63	1423	39.89	259	7.26
Mato Grosso do Sul	1	80	3.27	365	14.9	64	2.61	2	412	14.51	915	32.23	100	3.52
**Southeast**	57	6041	7.52	33,545	41.74	4677	5.82	107	19,776	22.06	72,741	81.15	8484	9.47
Espírito Santo	4	420	11.95	2128	60.54	213	6.06	5	900	21.91	3694	89.91	428	10.42
Minas Gerais	20	1752	8.94	9895	50.49	1147	5.85	32	4703	21.96	20,259	94.62	2183	10.2
Rio de Janeiro	13	1763	11.03	10,856	67.89	1705	10.66	17	4350	24.91	14,279	81.77	1937	11.09
São Paulo	20	2106	5.1	10,666	25.85	1612	3.91	53	9823	21.06	34,509	73.98	3936	8.44
**South**	13	1180	4.30	7019	25.63	979	3.57	28	6412	21.0	20,531	67.53	2178	7.16
Paraná	5	367	3.51	2103	20.13	300	2.87	13	3162	27.26	8711	75.11	945	8.15
Rio Grande do Sul	6	462	4.32	2632	24.61	403	3.77	13	1487	12.97	6084	53.06	700	6.1
Santa Catarina	7	351	5.62	2284	36.55	276	4.42	13	1763	24.02	5736	78.16	533	7.26
**Brazil**	98	9941	4.56	56,666	26.00	6960	3.19	218	42,837	17.78	144,040	59.80	16,089	6.68

*DA* Density of active students, *DF* Density of former students, *DV* Density of vacancies

^*^Per 100,000 population

Considering public and private schools (all schools), the mean and median of medical schools were 267 and 264 schools/year, respectively. The mean and median of vacancies in the period were 32,918 and 31,897 vacancies/year, respectively. The mean and median of active students were 125,586 and 114,599 students/year, respectively. The mean and median of former students were 17,992 and 16,959 students/year, respectively (data not shown in tables and/or figures).

As for public schools, the mean and median were 105 and 107 courses/year, respectively. The mean and median of vacancies were 9,276 and 9,487 vacancies/year, respectively. The mean and median of active students were 41,937 and 39,525, respectively. The mean and median of former students were 6,553 and 6,061 students, respectively.

As for private schools, the mean and median were 151 and 146 courses/year, respectively. The mean and median of vacancies were 23,642 and 22,410 vacancies/year, respectively. The mean and median of active students were 85,649 and 75,174, respectively. The mean and median of former students were 11,439 and 10,993, respectively (data not shown in tables and/or figures).

**Fig. 2:**
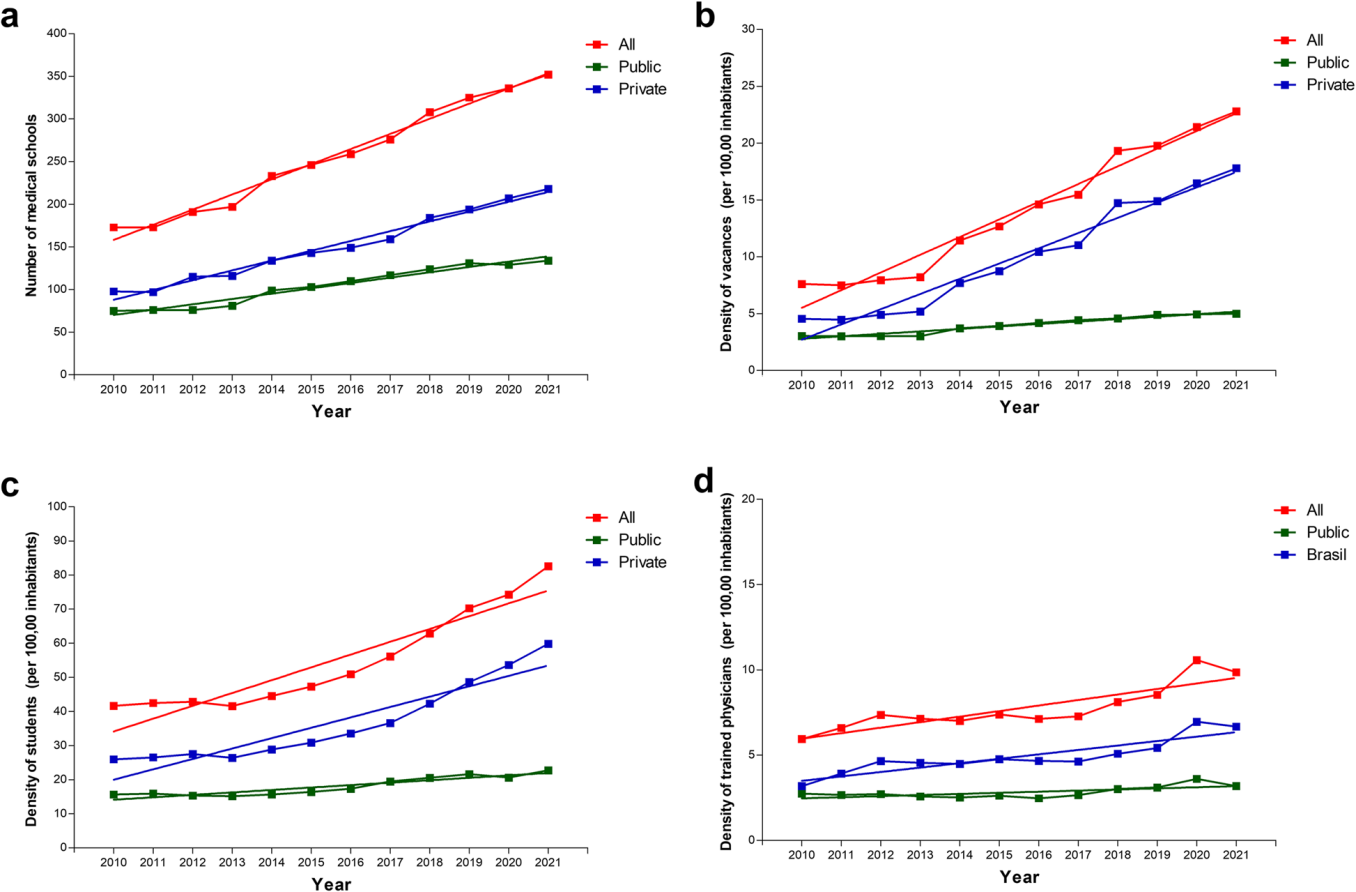
Temporal trend of medical education indicators in Brazil by public and private institutions, 2010–2021

The evolution of the indicators showed a greater medical education provision growth in private institutions. In all the years analyzed, private institutions provided the most vacancies and active and former students (Fig. 2).

In 2010, the southeast accounted for more than half of the number of vacancies (51.19%), active students (50.98%), and former students (55.1%), followed by the northeast, south, north, and Central-west. In 2021, the southeast increased the number of vacancies (55.24%), active students (63.65%), and former students (70.56%), followed by the northeast, south, north, and Central-west. The Southeast region is the most populous, so a higher proportion of these indicators is expected in this region when compared to the others (data not presented in tables and/or figures). When analyzing the vacancy density, in 2021, it appears that the highest densities are in the Central-West region (30.28 vacancies/100,000 inhabitants), followed by the Southeast (26.40 vacancies/100,000 inhabitants), South (26.34 vacancies/100 thousand inhabitants), Northeast (26.04 vacancies/100,000 inhabitants) and North (8.81 vacancies/100 thousand inhabitants). The pattern for density of active students was as follows: Southeast (102.29 active students/100,000 inhabitants), Central-West (99.59 active students/100,000 inhabitants), South (93.69 active students/100,000 inhabitants), Northeast (88.86 active students/ 100,000 inhabitants) and North (30.22 active students/100,000 inhabitants). For the density of former students, the following results are found between regions: Central-West (13.08 former students/100,000 inhabitants), Southeast (12.66 former students/100,000 inhabitants), South (10.77 former students/100,000 inhabitants), Northeast (10.09 former students/ 100,000 inhabitants) and North (3.36 former students/100,000 inhabitants) (Table 1).

In public schools, the southeast accounted for 36.73%, 37.34%, and 41.13% of the vacancies, active students, and former students, respectively. The second region with the highest contribution was the northeast, with 26.74%, 25.62%, and 24.11% of the vacancies, active students, and former students, respectively. The others were, in decreasing order, the south, north, and center-west. In 2021, the southeast accounted for 32.30%, 34.60%, and 37.37%, and the northeast accounted for 22.09%, 24.06%, and 22.49%, of the vacancies, active students, and former students, respectively. The other participating regions this year were, in order, the south, north, and center-west (data not shown in tables and/or figures). When analyzing the vacancy density for public schools, in 2021, it appears that the highest densities are in the Central-West region (15.19 vacancies/100,000 inhabitants), followed by the South (5.25 vacancies/100,000 inhabitants), Northeast (5.10 vacancies/100 thousand inhabitants), Southeast (4.34 vacancies/100,000 inhabitants) and North (2.60 vacancies/100 thousand inhabitants). The pattern for density of active students was as follows: Central-West (50.27 active students/100,000 inhabitants), South (26.17 active students/100,000 inhabitants), Northeast (25.31 active students/100,000 inhabitants), Southeast (25.31 active students/ 100,000 inhabitants) and North (12.06 active students/100,000 inhabitants). For the density of former students, the following results are found between regions: Central-West (6.43 former students/100,000 inhabitants), South (3.60 former students/100,000 inhabitants), Northeast (3.32 former students/100,000 inhabitants), Southeast (3.20 former students/ 100,000 inhabitants) and North (1.75 former students/100,000 inhabitants) (Table 2).

In private schools, the Northeast accounted for 60.77%, 59.20%, and 67.20% of the vacancies, active students, and former students, respectively. The second region with the highest contribution was the Northeast, with 15.91%, 16.58%, and 8.88% of the vacancies, active students, and former students, respectively. The others were, in decreasing order, the south, north, and center-west. In 2021, the southeast accounted for 46.17%, 50.50%, and 52.73%, and the northeast accounted for 25.43%, 22.96%, and 21.93%, of the vacancies, active students, and former students, respectively. This year, the south, north, and center-west participated consecutively. There is a greater proportion of private vacancies in the southeast, but there is a more equitable distribution in the other regions in 2021 (data not shown in tables and/or figures). When analyzing the vacancy density for private schools, in 2021, it appears that the highest densities are in the Southeast region (22.06 vacancies/100,000 inhabitants), followed by the Northeast (20.93 vacancies/100,000 inhabitants), South (21.0 vacancies/100 thousand inhabitants), Central-West (15.09 vacancies/100,000 inhabitants) and North (6.21 vacancies/100 thousand inhabitants). The pattern for density of active students was as follows: Southeast (81.15 active students/100,000 inhabitants), South (67.53 active students/100,000 inhabitants), Northeast (63.55 active students/100,000 inhabitants), Central-West (49.33 active students/ 100,000 inhabitants) and North (18.15 active students/100,000 inhabitants). For the density of former students, the following results are found between regions: Southeast (9.47 former students/100,000 inhabitants), South (7.16 former students/100,000 inhabitants), Northeast (6.78 former students/100,000 inhabitants), Central-West (6.65 former students/ 100,000 inhabitants) and North (1.51 former students/100,000 inhabitants) (Table 3).

There was a growing trend in vacancy density per 100,000 population in Brazil (APV: 29.3%; 95% CI: 23.8–35.0%). This occurred in public and private schools. However, the percentage increase was higher for private schools (APV: 37.6%; CI95%: 30.6–44.7%) than public ones (APV: 12.5%; 95% CI: 9.8–15.2%). Medical schools, regardless of type, showed an increasing trend in all 5 regions and the 26 states, except in Espírito Santo, which showed a stationary trend. As for public schools, all 5 regions and 17 (62.96%) of the 27 states showed an increasing trend, 7 states (25.92%) showed a stationary trend, and 3 (11.12%) a decreasing trend, represented by the states of Amazonas and Rondônia (north) and Espírito Santo (southeast). As for private schools, all 5 regions and 24 (88.88%) states showed an increasing trend, with 3 states showing a stationary trend (11.12%). No state showed a decreasing vacancy density in private schools (Table 4), like the results found for former student density (Table 5).

**Table 4:** Trend analysis of vacancy density/100,000 population by region, state, and type of institution (public or private) in the period 2010–2021

	**Total**					**Public**					**Private**				
**Region/State**	**APV (%)**	**LL**	**UL**	***p*-value**	**Trend**	**APV (%)**	**LL**	**UL**	***p*-value**	**Trend**	**APV (%)**	**LL**	**UL**	***p*-value**	**Trend**
**Center-West**	38.7	30.5	47.4	< 0.001	↑	45.8	38.4	53.6	< 0.001	↑	34.5	25.0	44.6	< 0.001	↑
Federal District	17.7	13.8	21.8	< 0.001	↑	-1.2	-2.5	0.2	0.085	−	29.8	22.7	37.4	< 0.001	↑
Goiás	61.3	49.7	73.8	< 0.001	↑	84.3	44.1	135.7	< 0.001	↑	41.1	22.6	62.4	< 0.001	↑
Mato Grosso	25.2	17.2	33.7	< 0.001	↑	23.6	4.1	46.8	0.022	↑	35.8	14.4	61.3	0.003	↑
Mato Grosso do Sul	31.4	20.0	44.0	< 0.001	↑	23.7	7.5	42.3	0.008	↑	37.2	20.8	55.9	< 0.001	↑
**Northeast**	35.5	30.0	41.2	< 0.001	↑	8.6	5.2	12.2	0.001	↑	52.0	43.9	60.7	< 0.001	↑
Alagoas	40.8	24.9	58.6	< 0.001	↑	10.9	8.3	13.7	< 0.001	↑	73.6	39.8	115.6	< 0.001	↑
Bahia	60.0	47.9	73.1	< 0.001	↑	13.9	-0.7	30.6	0.063	−	80.5	55.3	109.8	< 0.001	↑
Ceará	19.1	10.7	28.1	< 0.001	↑	1.4	-2.2	5.1	0.420	−	31.0	17.0	46.6	< 0.001	↑
Maranhão	31.7	24.4	39.5	< 0.001	↑	24.4	12.9	36.9	0.001	↑	38.0	29.3	47.3	< 0.001	↑
Paraíba	25.4	18.6	32.6	< 0.001	↑	0.2	-3.6	4.1	0.917	−	39.8	27.8	53.0	< 0.001	↑
Pernambuco	40.3	36.7	43.9	< 0.001	↑	54.9	15.8	107.0	0.008	↑	78.5	67.8	89.8	< 0.001	↑
Piauí	37.4	24.8	51.2	< 0.001	↑	16.7	10.9	22.8	< 0.001	↑	-12.2	-43.7	36.9	0.531	−
Rio Grande do Norte	26.4	14.7	39.4	< 0.001	↑	16.8	9.5	24.7	< 0.001	↑	33.7	12.3	59.1	0.004	↑
Sergipe	21.8	14.9	29.0	< 0.001	↑	8.1	0.5	16.3	0.041	↑	41.0	31.2	51.4	< 0.001	↑
**North**	29.5	26.0	11.9	< 0.001	↑	9.2	4.6	13.6	0.001	↑	49.8	33.7	21.9	< 0.001	↑
Acre	64.9	45.3	87.3	< 0.001	↑	16.2	3.4	30.7	0.018	↑	130.2	87.2	183.2	< 0.001	↑
Amazonas	17.4	11.1	24.1	< 0.001	↑	-3.0	-5.9	-0.1	0.046	↓	45.0	17.9	78.3	0.003	↑
Amapá	12.8	4.1	22.2	0.008	↑	12.8	4.1	22.2	0.008	↑	-	-	-	-	-
Pará	27.2	18.5	36.6	< 0.001	↑	-1.7	-8.4	5.4	0.600	−	65.7	49.8	83.2	< 0.001	↑
Rondônia	35.3	19.4	53.3	< 0.001	↑	-4.0	-7.1	-0.9	0.020	↓	41.7	23.6	62.4	< 0.001	↑
Roraima	25.8	15.1	37.6	< 0.001	↑	25.8	15.1	37.6	< 0.001	↑	-	-	-	-	-
Tocantins	22.1	14.2	30.5	< 0.001	↑	-27.1	-50.7	7.6	0.108	−	32.9	20.9	46.2	< 0.001	↑
**Southeast**	25.2	18.1	32.7	< 0.001	↑	8.3	4.1	12.6	0.001	↑	30.5	22.1	39.4	< 0.001	↑
Espírito Santo	-12.2	-32.2	13.6	0.290	−	-2.5	-3.6	-1.5	< 0.001	↓	-13.1	-34.5	15.3	0.298	−
Minas Gerais	19.4	15.5	23.5	< 0.001	↑	10.8	4.3	17.7	0.004	↑	24.1	19.0	29.3	< 0.001	↑
Rio de Janeiro	21.5	11.3	32.7	0.001	↑	0.3	-3.4	4.0	0.875	−	27.3	13.9	42.3	0.001	↑
São Paulo	33.8	25.8	15.8	< 0.001	↑	11.7	4.8	16.1	0.003	↑	40.4	32.2	15.6	< 0.001	↑
**South**	28.6	26.2	31.0	< 0.001	↑	7.4	3.8	11.0	0.001	↑	39.9	37.8	42.0	< 0.001	↑
Paraná	35.7	33.6	37.8	< 0.001	↑	9.8	7.5	12.2	< 0.001	↑	49.7	44.9	54.6	< 0.001	↑
Rio Grande do Sul	17.4	11.7	23.3	< 0.001	↑	7.0	4.5	9.5	< 0.001	↑	27.1	19.3	35.4	< 0.001	↑
Santa Catarina	28.5	22.7	34.6	< 0.001	↑	6.8	-9.3	25.8	0.394	−	35.2	27.3	43.6	0.394	−
**Brazil**	29.3	23.8	35.0	< 0.001	↑	12.5	9.8	15.2	< 0.001	↑	37.5	30.6	44.7	< 0.001	↑

*APV* annual percentage variation, *LL* lower limit, *UL* upper limit

**Table 5:** Trend analysis of active student density/100,000 population by region, state, and type of institution (public or private) in the period 2010–2021

	**Total**					**Public**					**Private**				
**Region/State**	**APV (%)**	**LL**	**UL**	***p*-value**	**Trend**	**APV (%)**	**LL**	**UL**	***p*-value**	**Trend**	**APV (%)**	**LL**	**UL**	***p*-value**	**Trend**
**Center-West**	24.5	19.3	30.0	< 0.001	↑	29.5	19.3	40.6	< 0.001	↑	20.8	17.9	23.8	< 0.001	↑
Federal District	10.0	6.5	13.6	< 0.001	↑	-1.0	-2.5	0.5	0.169	−	17.8	11.7	24.2	< 0.001	↑
Goiás	46.3	42.0	50.7	< 0.001	↑	58.5	29.8	93.4	< 0.001	↑	35.0	24.0	46.9	< 0.001	↑
Mato Grosso	15.0	9.3	20.9	< 0.001	↑	28.7	10.4	50.1	0.005	↑	7.2	-0.1	15.0	0.054	−
Mato Grosso do Sul	16.5	10.5	22.9	< 0.001	↑	18.6	9.3	28.7	0.001	↑	13.1	5.9	20.7	0.002	↑
**Northeast**	19.8	15.6	24.3	< 0.001	↑	7.9	5.0	10.8	< 0.001	↑	28.2	23.3	33.3	< 0.001	↑
Alagoas	33.7	24.2	43.9	0.001	↑	6.2	1.8	10.8	0.011	↑	154.9	110.0	209.3	< 0.001	↑
Bahia	31.7	21.4	43.0	< 0.001	↑	17.7	12.4	23.2	< 0.001	↑	41.4	26.7	57.8	< 0.001	↑
Ceará	11.4	9.8	13.1	< 0.001	↑	2.0	-3.0	7.3	0.405	−	17.9	13.9	22.0	< 0.001	↑
Maranhão	21.1	15.6	26.8	< 0.001	↑	18.3	12.9	24.1	< 0.001	↑	24.2	14.3	35.1	< 0.001	↑
Paraíba	15.6	12.5	18.7	< 0.001	↑	-3.8	-8.1	0.7	0.091	−	24.1	21.3	27.0	< 0.001	↑
Pernambuco	24.5	19.8	29.5	< 0.001	↑	5.1	3.4	6.9	< 0.001	↑	57.4	50.1	65.2	< 0.001	↑
Piauí	17.0	10.5	23.9	< 0.001	↑	13.9	11.3	16.7	< 0.001	↑	19.3	10.2	29.2	0.001	↑
Rio Grande do Norte	16.7	13.1	20.4	< 0.001	↑	15.5	11.5	19.6	< 0.001	↑	19.3	12.7	26.4	0.001	↑
Sergipe	23.4	19.2	27.7	< 0.001	↑	6.5	3.4	9.6	0.001	↑	80.7	54.7	111.2	< 0.001	↑
**North**	11.9	3.0	18.2	0.014	↑	2.0	-0.6	11.6	0.122	−	19.1	7.5	20.6	0.004	↑
Acre	38.4	29.3	48.1	< 0.001	↑	12.0	5.5	18.8	0.002	↑	167.6	123.6	220.3	< 0.001	↑
Amazonas	2.4	-3.4	8.7	0.391	−	-8.0	-14.3	-1.1	0.029	↓	19.3	0.7	41.3	0.045	−
Amapá	56.4	26.1	94.0	0.001	↑	56.4	26.1	94.0	0.001	↑	-	-	-	-	-
Pará	12.2	8.6	15.9	< 0.001	↑	-4.7	-8.7	-0.6	0.031	↓	44.4	37.6	51.6	< 0.001	↑
Rondônia	7.3	-2.1	17.7	0.123	−	-9.3	-15.4	-2.8	0.011	↓	9.7	-0.8	21.4	0.071	−
Roraima	18.6	11.7	25.9	< 0.001	↑	18.6	11.7	25.9	< 0.001	↑	-	-	-	-	-
Tocantins	4.3	-4.1	13.4	0.293	−	2.2	-2.7	7.3	0.357	−	5.4	-6.1	18.3	0.339	−
**Southeast**	12.9	8.4	17.6	< 0.001	↑	6.7	4.1	9.4	< 0.001	↑	15.0	9.5	20.8	< 0.001	↑
Espírito Santo	6.3	1.7	11.2	0.012	↑	-2.9	-4.9	-0.9	0.011	↓	8.0	2.3	14.0	0.011	↑
Minas Gerais	12.9	9.6	16.2	< 0.001	↑	10.4	8.6	12.2	< 0.001	↑	14.1	9.7	18.7	< 0.001	↑
Rio de Janeiro	3.5	-1.6	9.0	0.164	−	0.4	-0.7	1.6	0.43	−	4.5	-2.3	11.8	0.183	−
São Paulo	20.6	14.5	14.7	< 0.001	↑	8.4	2.8	14.8	0.008	↑	24.8	18.4	14.8	< 0.001	↑
**South**	15.8	11.7	20.0	< 0.001	↑	5.6	1.4	10.0	0.015	↑	22.5	18.1	27.0	< 0.001	↑
Paraná	22.9	20.0	25.9	< 0.001	↑	9.4	7.4	11.4	< 0.001	↑	31.6	29.7	33.5	< 0.001	↑
Rio Grande do Sul	11.1	7.1	12.9	< 0.001	↑	3.4	2.2	4.7	< 0.001	↑	17.6	10.9	24.7	< 0.001	↑
Santa Catarina	12.9	6.3	20.0	0.001	↑	5.8	-12.7	28.2	0.535	−	15.5	9.6	21.7	< 0.001	↑
**Brazil**	15.5	10.9	20.3	< 0.001	↑	8.7	4.6	13.0	0.001	↑	19.2	13.7	24.8	< 0.001	↑

*APV* annual percentage variation, *LL* lower limit, *UL* upper limit

Vacancy density, regardless of the type, showed an increasing trend in Brazil (AVP: 15.5%; 95% CI: 10.9–20.3). An increasing trend was also verified for public and private schools. Of the total, 24 (88.88%) states showed an increasing trend and 3 (11.12%) remained stationary. As for public institutions, 6 were stationary, 4 decreasing, and 17 increasing. Only the North presented a stationary trend, while the four other regions showed an increase. As for private institutions, 5 states showed a stationary trend, and 20 presented a growing trend. Two states (Amapá and Roraima) do not provide private education. All five regions showed an increasing trend (Table 5). Similar results, in general, were found for the density of former students (Table 6).

**Table 6:** Trend analysis of former students density/100,000 population by region, state, and type of institution (public or private) in the period 2010–2021

	**Total**					**Public**					**Private**				
**Region/State**	**APV (%)**	**LL**	**UL**	***p*-value**	**Trend**	**APV (%)**	**LL**	**UL**	***p*-value**	**Trend**	**APV (%)**	**LL**	**UL**	***p*-value**	**Trend**
**Center-West**	18.5	6.6	31.5	0.005	↑	17.8	1.4	36.8	0.037	↑	18.2	6.8	30.8	0.005	↑
Federal District	3.5	-5.5	13.4	−	−	-4.3	-13.1	5.3	0.334	−	8.3	-4.4	22.8	0.189	−
Goiás	45.6	31.5	61.3	< 0.001	↑	29.4	4.6	60.1	0.024	↑	50.2	38.4	63.0	< 0.001	↑
Mato Grosso	15.1	-2.5	35.8	0.091	−	47.1	33.0	62.7	< 0.001	↑	1.7	-17.4	25.1	0.864	−
Mato Grosso do Sul	10.6	-0.9	23.4	0.072	−	10.3	-5.4	28.5	0.189	−	26.5	-5.7	69.7	0.108	−
**Northeast**	17.5	10.5	24.9	< 0.001	↑	5.7	0.8	10.8	0.028	↑	32.1	14.6	52.1	0.002	↑
Alagoas	18.1	-7.5	50.8	0.164	−	0.8	-3.0	4.6	0.669	−	45.7	-10.1	136.3	0.117	−
Bahia	24.1	8.3	42.2	0.006	↑	5.6	-2.2	14.0		−	43.5	10.4	86.5	0.013	↑
Ceará	12.4	6.3	18.8	0.001	↑	4.3	-2.1	11.2	0.174	−	26.0	6.2	49.5	0.014	↑
Maranhão	13.0	3.3	23.5	0.013	↑	19.4	2.5	39.1	0.028	↑	0.9	-9.6	12.7	0.856	−
Paraíba	18.1	9.2	27.8	< 0.001	↑	1.0	-8.3	11.1	0.830	−	30.2	17.3	44.6	< 0.001	↑
Pernambuco	15.3	9.4	21.5	< 0.001	↑	0.7	-6.8	8.8	0.844	−	35.3	21.1	51.1	< 0.001	↑
Piauí	6.3	-3.4	16.9	0.019	−	-4.1	-16.8	10.6	0.532	−	13.7	-0.1	29.1	0.051	−
Rio Grande do Norte	24.2	5.4	46.3	0.016	↑	14.9	1.8	29.6	0.030	↑	30.0	1.0	67.2	0.045	↑
Sergipe	26.8	18.3	36.0	< 0.001	↑	11.8	4.3	20.0	0.013	↑	40.4	23.8	59.3	< 0.001	↑
**North**	6.7	0.1	16.1	0.049	↑	3.9	1.0	11.9	0.013	↑	13.5	-4.8	29.8	0.145	−
Acre	8.0	-24.1	537	0.642	−	0.3	-20.4	26.4	0.979	−	43.6	-8.8	126.1	0.110	−
Amazonas	-3.4	-14.7	9.4	0.552	−	2.4	-6.4	12.0	0.579	−	-13.3	-33.2	12.5	0.254	−
Amapá	-36.7	-55.8	-9.3	0.038	↓	24.8	-9.8	72.7	0.164	−	-	-	-	-	-
Pará	4.7	0.8	8.7	0.024	↑	-7.8	-14.9	0.1	0.050	−	21.4	7.8	36.8	0.005	↑
Rondônia	6.6	-7.3	22.5	0.338	−	-6.3	-11.8	-0.6	0.036	↓	10.1	-8.0	31.8	0.267	−
Roraima	10.6	-19.0	50.9	0.495	−	30.5	-6.8	82.7	0.112	−	-	-	-	-	-
Tocantins	11.0	-0.2	23.4	0.057	−	23.0	-6.6	62.0	0.129	−	4.6	-2.2	11.8	0.173	−
**Southeast**	6.9	2.8	11.2	< 0.001	↑	2.0	-4.0	8.4	0.486	−	8.7	4.1	13.6	0.002	↑
Espírito Santo	8.5	5.0	12.1	< 0.001	↑	-0.3	-8.1	8.2	0.943	−	10.6	3.6	18.1	0.007	↑
Minas Gerais	9.9	5.5	14.4	< 0.001	↑	7.8	4.7	11.0	< 0.001	↑	11.0	4.6	17.8	0.003	↑
Rio de Janeiro	-1.5	-4.3	1.3	0.238	−	0.2	-5.0	5.6	0.934	−	-2.3	-5.7	1.2	0.175	−
São Paulo	10.8	2.4	17.8	0.017	↑	-0.5	-8.7	18.6	0.898	− ↑	15.3	8.7	15.5	< 0.001	↑
**South**	8.7	4.6	12.9	0.001	↑	2.2	-3.5	8.3	0.486	−	13.2	7.9	18.8	< 0.001	↑
Paraná	15.7	11.4	20.1	< 0.001	↑	4.0	1.1	7.0	0.011	−	25.0	19.9	30.3	< 0.001	↑
Rio Grande do Sul	5.0	0.5	9.8	0.033	↑	2.2	-2.8	7.5	0.354	−	8.4	2.8	14.2	0.007	↑
Santa Catarina	5.2	0.6	10.1	0.033	↑	3.9	-13.5	24.9	0.654	−	8.0	-3.9	21.4	0.177	−
**Brazil**	10.1	5.6	14.9	0.001	↑	4.0	-1.5	10.5	0.134	−	13.8	7.3	20.6	< 0.001	↑

*APV* annual percentage variation, *LL* lower limit, *UL* upper limit

**Fig. 3:**
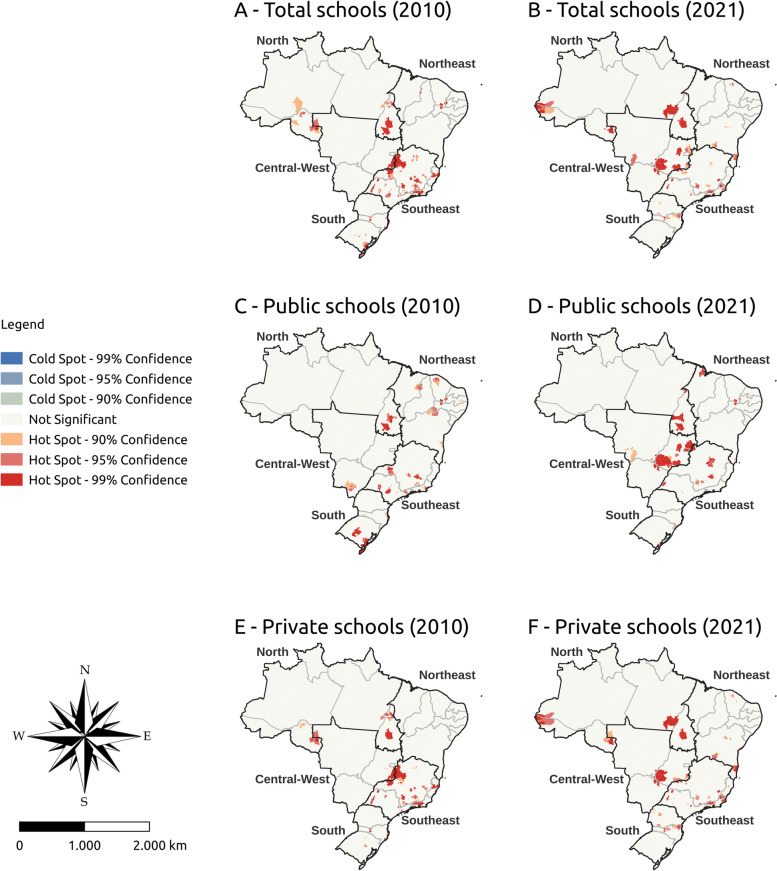
Vacancy density hot spot in undergraduate medical programs in Brazil, 2010 and 2021

**Fig. 4:**
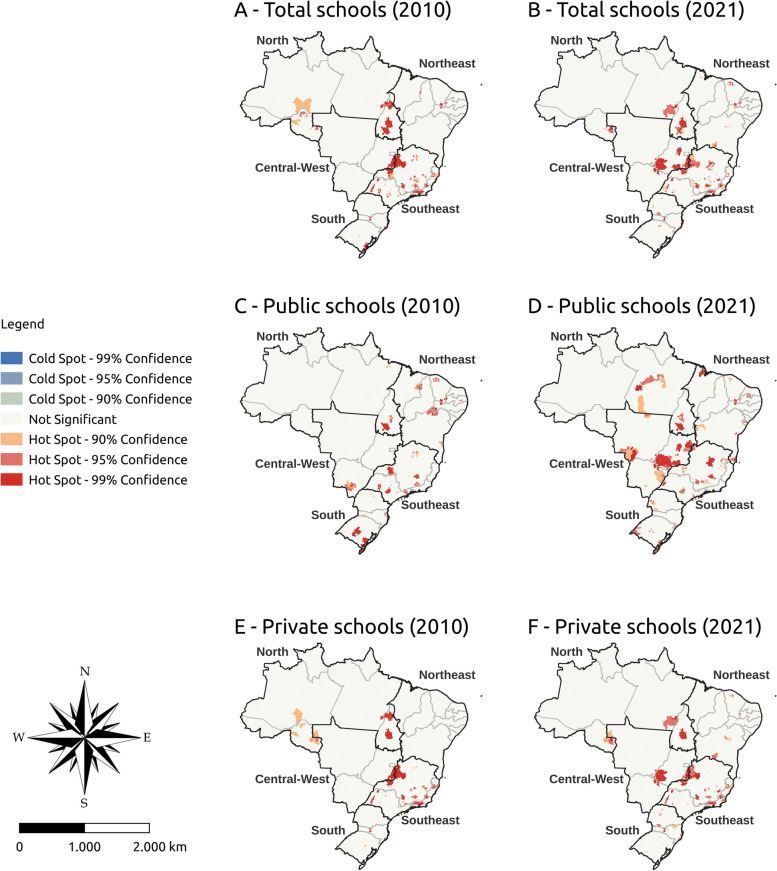
Active students density hot spot in undergraduate medical programs in Brazil, 2010 and 2021

**Fig. 5:**
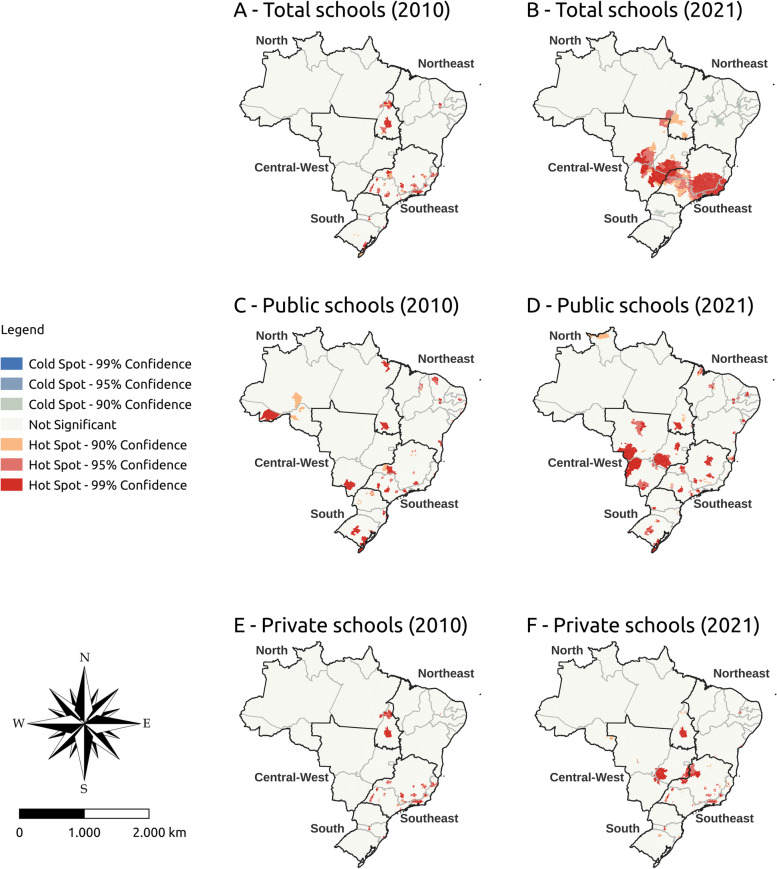
Former students density hot spot in undergraduate medical programs in Brazil, 2010 and 2021

Figures 3, 4, and 5 show the vacancy density hot spots and active and former students density indicators in Brazil in 2010 and 2021, respectively. Cold Spots were not found for any analyzed indicator. The analyses were stratified by public and private institutions.

In 2010, most vacancy density hot spots were concentrated in Minas Gerais, São Paulo, and Rio de Janeiro (southeast), regardless of the type of school. In 2021, vacancy density showed a spatial distribution in the center-west, more specifically in Goiás, and some states in the north. Public schools also showed a spatial distribution of hot spots from 2010 to 2021 in the center-west (specifically Goiás) and north (specifically Tocantins). Private schools had the hot spots concentrated in the southeast in 2010, with greater spatial distribution in the north and center-west in 2021 (Fig. 3).

The density of active students showed a greater number of hot spots in the center-west and northeast in 2021 with greater maintenance in the southeast. This pattern was similar in public schools, with a hot spot increase in the center-west and north. As for private schools, the hot spots remained the same in the southeast (more specifically in Minas Gerais) between 2010 and 2021. New hot spots appeared in private schools in the center-west and north (Fig. 4).

Former students density hot spots with 99% significance were concentrated in the southeast and center-west in 2021. Public schools showed greater spatial distribution of this indicator between 2010 and 2021, with a higher hot spot concentration in the center-west and southeast. Private schools had the largest number of hot spots also concentrated in the southeast and center-west (Fig. 5).

## Discussion

This study analyzed the trend of medical education supply indicators disaggregated by public and private education, states, and regions. The number of programs, vacancies, and active and former students increased during the period, especially in private institutions. There was a growing trend in the density of vacancies and active and former students in all five regions and most states in Brazil. At the end of 2021, most vacancies and active and former students were still concentrated in the southeast. Medical education remains uneven in Brazil, with low medical education provisions in regions with lower socioeconomic development, academic structure, and health services, represented by the north and northeast. Hot Spot Analysis identified vacancy density and active and former students hot spots predominantly in the southeast, center-west, and north. Despite the importance and relevance of disaggregated analyses by state and region and the evaluation of spatial clusters for these indicators, there is a lack of recent literature on such topics. This study aggregates these data.

There was a growing trend in medical education indicators in the public and private sectors, but this increase was greater in private institutions. This result corroborates those of previous studies showing that private HEIs provide the largest number of medical schools and the highest percentage of medical vacancies and enrollments in almost all Brazilian states [11]. This research corroborates other studies, showing the privatization of higher education in the country [2, 6, 19]. In Brazil, a study showed a simultaneous change in the number of public schools in relation to private schools over time. Most schools were publicly funded until 2006 when private funding began to prevail and continued with an increasing trend in the following years [33].

Multiple inter-sectoral programs and policies in the fields of health and education have affected medical education over time, boosting the growth of medical education in Brazil [34]. Some of these programs and policies increased the number of vacancies in the public sector and others in the private sector.

In the healthcare sector, the PISUS (1993) aimed at promoting health and retaining physicians and other health professionals in the interior, with an adequate physical structure for professional performance and payment for production through the transfer of resources by the Brazilian Ministry of Health [13, 35]. This program reached 398 cities between 1993–1994 [36]. Another program was the PITS (2001), which encouraged the allocation of qualified health professionals to cities with smaller medical workforces and far from capitals and large urban centers, also working to expand PHC coverage in the country [14, 35]. In the operating period (2001–2004), 300 cities were covered [36]. In 2007, Telehealth was implemented, a strategy of the Ministry of Health National Policy for Permanent Education (in Portuguese, *Política Nacional de Educação Permanente do Ministério da Saúde*), aimed at the training and development of human resources in health and PHC qualification and management. Telehealth has currently integrated teaching and practice through tele-assistance and tele-education. It has been used to increase the retention of professionals in remote and more vulnerable areas [37, 38]. In 2011, PROVAB was instituted to provide PHC teams with health professionals in remote and more vulnerable areas [39]. The professionals received a federal government grant, supervision, and the opportunity to participate in a PHC specialization program. At the end of the year, physicians received a 10% score on their grades in medical residency programs [16]. In 2015, PROVAB was integrated into the PMM [35]. The actions of previous programs and policies were aimed at strengthening the SUS health workforce.

The expansion of HEIs and vacancies in medical programs increased the number of vacancies and scholarships in medical residency programs. In 2009, the Ministries of Health and Education established the National Program to Support the Training of Specialist Doctors in Strategic Areas (in Portuguese, *Programa Nacional de Apoio à Formação de Médicos Especialistas em Áreas Estratégicas* [Pró-Residência]), intending to promote scholarships, training specialists in priority areas, and open new medical residency programs considering SUS regional needs [40]. In line with the previous policy, in 2021, the Ministry launched the National Plan for Strengthening Health Residencies, that is, a set of strategic actions to promote the appreciation and qualification of residents, faculty, and managers, contributing to the qualified training of health professionals, institutionally supporting these programs, and expanding the number of residency programs in health with grants financed by the Ministry of Health in priority regions (center-west, northeast, and north) [41]. This program and plan are in operation and were responsible for promoting vacancies in residency programs in public and private institutions, increasing the number of health graduate program vacancies in Brazil.

One of the health programs that contributed the most to the expansion of higher education was the PMM, implemented in 2013. This program expanded medical education in the private sector [11]. The PMM increased vacancies and expanded the number of trained medical professionals, especially in the private sector, seeking alternatives for allocating physicians to interior cities in order to increase the fixation and homogeneity of workforce spatial distribution in Brazil [42]. This program was also responsible for expanding residency programs in family and community medicine to strengthen PHC. It brought over 14,000 foreign physicians in a short time and created 11,400 new vacancies in medical schools over 3–5 years [19]. Evidence shows that the PMM has significantly expanded medical education in Brazil, especially in the private sector, including a more decentralized provision of programs in smaller cities and outside the capitals and metropolitan regions [11, 43].

Simultaneously, other educational policies contributed to the expansion of medical education in Brazil. In 2001, the Ministry of Health implemented the Student Financing Fund (in Portuguese, *Fundo de Financiamento Estudantil*) to fund students in undergraduate medical programs in private universities. It also amortized the loans of physicians working in PHC teams in areas with a low number of physicians. This strategy expanded the medical workforce in Brazil [35, 44]. The University for All Program (in Portuguese, *Programa Universidade para Todos* [PROUNI]) was created in 2004 to provide partial or full scholarships for undergraduate courses in private institutions [45]. These programs increased the access of medical students to higher education in the private sector. These policies, driven by government incentives, also increased the number of large corporate groups and conglomerates to provide private medical education, exploring this sector from a marketing point of view [46].

In 2007, REUNI was instituted to expand access and permanence in undergraduate courses, reducing dropout rates and idle vacancies and increasing admission vacancies in federal public universities [15]. In 2013, the National Policy for the Expansion of Medical Schools in Federal HEIs (in Portuguese, *Política Nacional de Expansão das Escolas Médicas das Instituições Federais de Educação Superior*) (2013) created medical programs and expanded vacancies in existing undergraduate programs in federal public universities. In 2022 alone, this policy provided 2,016 vacancies in medical programs, all in priority regions and cities outside of the capitals and metropolitan regions [17, 47]. The REUNI and the National Policy for the Expansion of Medical Schools of Federal HEIs increased the provision of medical education in federal universities but not enough to match the number of vacancies in private education institutions [11]. Also, limited financial resources, growing demand for health professionals and business opportunities for big players educational groups, favor this difference in growth between the number of public and private schools [10]. Furthermore, it appears that the density of vacancies per 100,000 inhabitants for public schools is almost unchanged and has only followed, to a certain extent, population growth, unlike the density of vacancies per 100,000 inhabitants in private schools. It should be noted that it is up to the public sector to guarantee a human resources training policy to improve the health of the population throughout the national territory. In fact, the present study shows inequalities between regions in the indicators of medical education supply in the country. These results increase inequalities in medical education, becoming increasingly distant from meeting the population's health needs, especially in the most vulnerable regions such as the North and Northeast.

The phenomenon of medical education privatization, with a greater number of vacancies in private courses, can be seen as a limiting factor in reducing inequalities. Despite the improvements generated by higher education financing programs (FIES and PROUNI), the high investment required to pay medical school fees or financing installments reduces the chances of lower-income students enrolling in medical programs. In addition, most private courses are located in large urban centers [11], hindering the improvement of health access, especially in PHC, and the number of physicians in more vulnerable areas. These factors increase the probability of persistent inequalities in the labor force in the regions as people trained in capitals and urban centers tend to maintain their employment relationship in the same place they graduated from [48]. The results of this study show that only part of the vacancies is provided by public institutions. This indicates that some public policies to increase access to public higher education can be improved. Comprehensive actions focusing on the equitable distribution of vacancies in public and private institutions are essential for reversing inequalities [49].

This debate on the expansion of vacancies must be implemented by evaluating the quality of the education provided through accreditation systems. These systems ensure that recently graduated physicians are ready to continue their education or start their professional practice. In 2020, data from the World Directory of Medical Schools showed that only 49% of countries had access to undergraduate accreditation with specific medical standards [4]. Brazil has two accreditation systems [4]. The first one is the National Higher Education Assessment System (in Portuguese, *Sistema Nacional de Avaliação de Educação Superior* [SINAES]) of the Ministry of Education, which evaluates institutions, programs, and student performance considering evaluative aspects such as teaching quality, research, extension, social responsibility, management, and the faculty. The data are used to guide educational institutions and support public policies [50]. The second one is the Accreditation System for Medical Courses in Brazil (in Portuguese, *Sistema de Acreditação dos Cursos de Medicina no Brasil* [SAEME]), created by the Federal Council of Medicine in 2016 as a strategy for qualifying medical training in the country. It is an evaluation process based on a set of quality indicators, identifying education weaknesses and areas of excellence [51]. INEP data showed that most medical programs in the country are classified as medium quality (grade 3), on a 1–5 scale. In addition, no medical school in Brazil obtained the maximum grade over three consecutive evaluations [9].

This study showed spatial inequalities in the distribution of vacancies and active and former students between regions in public and private institutions. In 2021, most hot spots were found in the southeast, northern, and center-west. This result ratifies the unequal provision of medical education and workforce in Brazil. This imbalanced medical workforce and poor spatial distribution affect several countries. In addition, the lack of professionals in regions with greater vulnerability, such as rural and poor areas, is a worldwide problem, including in Brazil [20]. This imbalance reduces access to health services and the universal coverage of health care, as established by Target 3.8 of SDG 3 (Health and Well-being) [21, 35]. Evidence shows that the poor geographic distribution of PHC professionals and specialists is primarily caused by the growing demand for professionals due to the increased number of health institutions, especially in PHC, a low number of new physicians from medical programs compared to existing demand and growing needs, and a low level of development in the cities, worse living conditions, low-quality medical residency programs and practice scenarios compared to capitals, and poor working conditions, among other factors [35]. For example, in Brazil, small cities have a ratio of 0.63 physicians/1,000 population, which is almost five times lower than that found in cities with more than 500,000 population. These cities have the lowest socioeconomic development levels and, therefore, the lowest access to and coverage of health services. Medical demography data from 2020 show that the regions in the center-west and southeast have rates of 2.74, and the south has 3.15 physicians/1,000 population, respectively. However, the northeast and north presented ratios of 1.69 and 1.30 physicians/1,000 population, respectively [10].

This study presented some limitations. We cannot rule out the underestimation or overestimation of the indicators due to variable recording failures. However, everyone filling in the information underwent rigorous training. Only total vacancies were analyzed, but not new vacancy density trends in the year, as these data were separated in the database only after 2014. Therefore, the analysis of new vacancies in medical programs is another limitation. Trends did not undergo sensitivity analysis by types of evaluation concept, which could contribute to understanding whether the expansion of medical education was taking place in better or lesser quality programs. However, this study has strong points, which include its national coverage, disaggregation by public and private institutions and states and regions, and the spatial analysis of hot spots in the distribution of medical education.

## Conclusions

In conclusion, indicators of medical education provision increased in Brazil, especially in the private sector. However, this provision remains low in less developed regions. Hot spots were found in the southeast, center-west, and north in 2021. The results show inequities in the provision of medical education in Brazil, despite its temporal increase. These inequalities and imbalances in the supply of medical education between public and private institutions, the evidenced regional inequalities and the slow pace of expansion of vacancies in the public sector compromise public health policies, weakening the SUS, especially for not being able to train enough doctors for areas priorities, such as Primary Health Care and operating in more vulnerable areas. The data from this study may support medical workforce planning policies in Brazil. The expansion of vacancies in the public and private sectors must consider program quality, geographic distribution, medical workforce inequalities, regional health needs, and access to health services by the population, among other aspects. The expansion of vacancies in public institutions is essential to ensure equity between public and private education, training doctors for the SUS and should be the target of public policies. Finally, new studies must be carried out, especially those that investigate the reasons for the low expansion of vacancies in the public sector, the impact of training, mainly private, on the quality of medical training, in addition to analyzes that investigate the contextual determinants of the supply of vacancies in the sectors public and private (for example: per capita income, development index, academic structure, attractiveness indicators, among others).

## Data Availability

The datasets generated and/or analyzed during the current study are available in the INEP repository [https://www.gov.br/inep/pt-br/acesso-a-informacao/dados-abertos/microdados/censo-da-educacao-superior] and IBGE repository [https://www.ibge.gov.br/estatisticas/sociais/populacao/9103-estimativas-de-populacao.html?=&t=resultados].

## Abbreviations

PHC: Primary Health Care
SE: Standard error
FIES: Student Financing Fund (in Portuguese, *Fundo de Financiamento Estudantil*)
IBGE: Brazilian Institute of Geography and Statistics (in Portuguese, *Instituto Brasileiro de Geografia e Estatística*)
95% CI: 95% Confidence interval
HDI: Human Development Index
HEI: Higher Education Institutions (in Portuguese, *Instituições de Ensino Superior*)
INEP: Anísio Teixeira National Institute of Educational Studies and Research (in Portuguese, *Instituto Nacional de Estudos e Pesquisas Educacionais Anísio Teixeira*)
LDB: Law of Educational Guidelines and Bases (in Portuguese, *Lei de Diretrizes e Bases da Educação*)
SDG: Sustainable Development Goals
GDP: Gross Domestic Product
PAHO: Pan American Health Organization
PITS: Health Work Growth in Interior Cities Program (in Portuguese, *Programa de Interiorização do Trabalho em Saúde*)
PMM: More Doctors Program (in Portuguese, *Programa Mais Médicos*)
POVAB: Valorization Program for Primary Care Professionals (in Portuguese*, Programa de Valorização dos Profissionais da Atenção Básica*)
Pró-Residência: National Program to Support the Training of Specialist Doctors in Strategic Areas (in Portuguese, *Programa Nacional de Apoio à Formação de Médicos Especialistas em Áreas Estratégicas*)
PROUNI: University for All Program (in Portuguese, *Programa Universidade para Todos*)
REUNI: Support Program for the Restructuring and Expansion of Federal Universities (in Portuguese, *Programa de Apoio à Reestruturação e Expansão das Universidades Federais*)
SAEME: Accreditation System for Medical Courses in Brazil (in Portuguese, *Sistema de Acreditação dos Cursos de Medicina no Brasil*)
SINAES: National Higher Education Assessment System (in Portuguese, *Sistema Nacional de Avaliação de Educação Superior*)
SUS: Unified Health System (in Portuguese, *Sistema Único de Saúde*)
APV: Annual Percentage Variation
WHO: World Health Organization

## References

[1] Amaral JLG (2016). O exame terminal salvaguarda a Medicina. Rev da Assoc Paul Med.

[2] de Oliveira BLCA, Lima SF, Pereira MUL, Pereira Júnior GA (2019). Evolução, distribuição e expansão dos cursos de medicina no Brasil (1808–2018). Trab Educ e Saúde.

[3] Rizwan M, Rosson N, Tackett S, Hassoun H (2018). Opportunities and challenges in the current era of global medical education. Int J Med Educ.

[4] Bedoll D, van Zanten M, McKinley D. Global trends in medical education accreditation. Hum Resour Health. 2021;19:70. 10.1186/s12960-021-00588-x10.1186/s12960-021-00588-x . 10.1186/s12960-021-00588-x PMC8136216 34016122

[5] Mattei J, Malik V, Wedick NM, Hu FB, Spiegelman D, Willett WC (2015). Reducing the global burden of type 2 diabetes by improving the quality of staple foods: The Global Nutrition and Epidemiologic Transition Initiative. Global Health..

[6] Pereira DVR, Fernandes D de LR, Mari JF, Lage AL de F, Fernandes APPC. Cartografia das escolas médicas: a distribuição de cursos e vagas nos municípios brasileiros em 2020. Rev Bras Educ Med. 2021;45(1):1–10. 10.1590/1981-5271v45.1-20200282 .

[7] Presidência da República, Casa Civil, Subchefia para Assuntos Jurídicos. Lei número 12.871, de 22 de outubro de 2013. Institui o Programa Mais Médicos, altera as Leis número 8.745, de 9 de dezembro de 1993, e n^o^ 6.932, de 7 de julho de 1981, e dá outras providências. Brasília-DF: Presidência da República. 2013. Available from: https://www.planalto.gov.br/ccivil_03/_ato2011-2014/2013/lei/l12871.htm. Cited 19 Feb 2023.

[8] Presidência da República, Secretaria-Geral, Subchefia para Assuntos Jurídicos. Lei número 13.958 de 18 de dezembro de 2019. Institui o Programa Médicos pelo Brasil, no âmbito da atenção primária à saúde no Sistema Único de Saúde (SUS), e autoriza o Poder Executivo federal a instituir serviço social autônomo denominado Agência para o. Presidência da República. 2019. Available from: https://www.planalto.gov.br/ccivil_03/_ato2019-2022/2019/lei/l13958.htm. Cited 9 Jun 2023.

[9] Santos Júnior CJ dos, Misael JR, Trindade Filho EM, Wyszomirska RM de AF, Santos AA dos, Costa PJM de S. Expansão de vagas e qualidade dos cursos de Medicina no Brasil: “Em que pé estamos?” Rev Bras Educ Med. 2021;45(2):1–10. 10.1590/1981-5271v45.2-20200523 .

[10] Scheffer M. Demográfia médica no Brasil 2020. São Paulo-SP: Faculdade de Medicina da Universidade de São Paulo. São Paulo: Departamento de Medicina Preventiva da Faculdade de Medicina da USP; Conselho Federal de Medicina; 2020:312. Available from: https://www.fm.usp.br/fmusp/conteudo/DemografiaMedica2020_9DEZ.pdf. Cited 19 Feb 2023.

[11] Scheffer MC, Dal Poz MR (2015). The privatization of medical education in Brazil: Trends and challenges. Hum Resour Health.

[12] Hone T, Powell-Jackson T, Pacheco Santos LM, de Sousa SR, de Oliveira FP, Sanchez MN, et al. Impact of the Programa Mais Medicos (More Doctors Programme) on primary care doctor supply and amenable Mortality. BMC Health Serv Res. 2020;20:873. 10.1186/s12913-020-05716-2. 10.1186/s12913-020-05716-2 PMC7491024 32933503

[13] Ministério da Saúde. Programa de Interiorização do Sistema Único de Saúde - PISUS. Brasília-DF: Ministério da Saúde. 1993:17. Available from: https://pesquisa.bvsalud.org/portal/resource/pt/lil-128275. Cited 9 Jun 2023.

[14] Presidência da República, Casa Civil, Subchefia para Assuntos Jurídicos. Decreto número 3.745, de 5 de fevereiro de 2001. Institui o Programa de Interiorização do Trabalho em Saúde. Brasília-DF: Ministério da Saúde. 2001:2. Available from: https://www.planalto.gov.br/ccivil_03/decreto/2001/D3745.htm. Cited 19 Feb 2023.

[15] Presidência da República, Casa Civil, Subchefia para Assuntos Jurídicos. Decreto número 6.096, de 24 de abril de 2007. Institui o Programa de Apoio a Planos de Reestruturação e Expansão das Universidades Federais - REUNI. Brasília-DF: Presidência da República. 2007:2. Available from: https://www.planalto.gov.br/ccivil_03/_ato2007-2010/2007/decreto/d6096.htm.Cited 19 Feb 2023.

[16] Ministério da Saúde. Portaria interministerial número 2.087, de 1 de setembro de 2011. Institui o Programa de Valorização do Profissional da Atenção Básica. Brasília-DF: Ministério da Saúde. 2011 . Available from: https://bvsms.saude.gov.br/bvs/saudelegis/gm/2011/pri2087_01_09_2011_rep.htmlCited 19 Feb 2023.

[17] Ministério da Educação. Portaria normativa número 015, de 22 de julho de 2013. Institui a Política Nacional de Expansão das Escolas Médicas das Instituições Federais de Educação Superior - IFES. Brasília-DF: Ministério da Educação. 2013, 2 p. Available from: http://portal.mec.gov.br/index.php?option=com_docman&view=download&alias=14699-portaria-norm-n015-instititui-politica-nac-ifes&category_slug=novembro-2013-pdf&Itemid=30192. Cited 19 Feb 2023.

[18] Santos LMP, Oliveira A, Trindade JS, Barreto IC, Palmeira PA, Comes Y (2017). Implementation research: towards universal health coverage with more doctors in Brazil. Bull World Health Organ.

[19] Figueiredo AM, McKinley DW, Massuda A, Azevedo GD (2021). Evaluating medical education regulation changes in Brazil: workforce impact. Hum Resour Health..

[20] World Health Organization. Global strategy on human resources for health: Workforce 2030 Geneva: World Health Organization. 2016: 64. Available from: https://apps.who.int/iris/bitstream/handle/10665/250368/9789241511131-eng.pdf. Cited 19 Feb 2023.

[21] United Nations. The Sustainable Development Goals Report 2022. Geneva: United Nations. 2022:68. Available from: https://unstats.un.org/sdgs/report/2022/. Cited 19 Feb 2023.

[22] Instituto Brasileiro de Geografia e Estatística. IBGE Cidades. Brasília-DF: IBGE, 2022. Available from: https://cidades.ibge.gov.br/brasil/panorama. Cited 27 Feb 2023.

[23] United Nations Development Programme. Human Development Report 2021/2022 - Uncertain Times, Unsettled Lives: Shaping our Future in a Transforming World. New York, NY: United Nations Development Programme. 2022:320. Available from: https://hdr.undp.org/content/human-development-report-2021-22 . Cited 27 Feb 2023.

[24] Instituto Nacional de Estudos e Pesquisas Educacionais Anísio Teixeira. Censo da Educação Superior: microdados do Centro da Educação Superior. Brasília-DF: Instituto Nacional de Estudos e Pesquisas Educacionais Anísio Teixeira. 2019:1. Available from: https://www.gov.br/inep/pt-br/acesso-a-informacao/dados-abertos/microdados/censo-da-educacao-superior. Cited 19 Feb 2023.

[25] Instituto Nacional de Estudos e Pesquisas Educacionais Anísio Teixeira. Metodologia de Coleta do Censo da Educação Superior - 2019 [Internet]. Brasília-DF: Instituto Nacional de Estudos e Pesquisas Educacionais Anísio Teixeira. 2020:64. Available from: http://portal.inep.gov.br/informacao-da-publicacao/-/asset_publisher/6JYIsGMAMkW1/document/id/6970659. Cited 19 Feb 2023.

[26] Presidência da República, Casa Civil, Jurídicos S para A. Lei número 9.394 de 20 de dezembro de 1996. Estabelece as diretrizes e bases da educação nacional. [Internet]. Brasília-DF: Presidência da República. 1996: 28. Available from: https://www.planalto.gov.br/ccivil_03/leis/l9394.htm . Cited 19 Feb 2023.

[27] Instituto Brasileiro de Geografia e Estatística. Estimativas da população [Internet]. Brasília-DF: Instituto Brasileiro de Geografia e Estatística. 2022:1. Available from: https://www.ibge.gov.br/estatisticas/sociais/populacao/9103-estimativas-de-populacao.html?=&t=o-que-e .Cited 23 Feb 2023.

[28] R Core Team. R: A language and environment for statistical computing . Vienna, Austria: R Foundation for Statistical Computing. 2022. Available from: https://www.r-project.org/. Cited 18 Feb 2023.

[29] Antunes JLF, Cardoso MRA. Using time series analysis in epidemiological studies. Epidemiol e Serviços Saúde. 2015;24(3):565–76. 10.5123/s1679-49742015000300024.

[30] Getis A, Ord JK. The analysis of spatial association by use of distance statistics. Geogr Anal. 1992;24(3):180–206. 10.1111/j.1538-4632.1992.tb00261.x.

[31] Vissoci JRN, Rocha TAH, da Silva NC, de Sousa Queiroz RC, Thomaz EBAF, Amaral PVM (2018). Zika virus infection and microcephaly: Evidence regarding geospatial associations. PLoS Negl Trop Dis.

[32] Environmental Systems Research Institute. ArcGIS 10.3. Redlands: Environmental Systems Research Institute. 2014. Available from: https://www.esri.com/arcgis-blog/products/3d-gis/3d-gis/arcgis-10-3-the-next-generation-of-gis-is-here/ .Cited 23 Feb 2023.

[33] Franco T de AV, Dal Poz MR. A privatização de instituições de ensino superior privadas na formação em saúde no Brasil. Trab. Educ. Saúde. 2018;16(3):1017–37.

[34] Santos RA dos, Nunes M do PT. Medical education in Brazil. Med Teach. 2019;41(10):1106–11. Available from: 10.1080/0142159X.2019.163695510.1080/0142159X.2019.1636955 . 10.1080/0142159X.2019.1636955 31282823

[35] de Oliveira APC, Gabriel M, Dal Poz MR, Dussault G (2017). Challenges for ensuring availability and accessibility toin health care services under Brazil’s unified health system (SUS). Cienc e Saude Coletiva.

[36] Carvalho MS de. Programa de Valorização dos Profissionais da Atenção Básica: um olhar implicado sobre sua implantação [Internet]. Brasília-DF: Universidade de Brasília. 2013:167. Available from: https://repositorio.unb.br/bitstream/10482/13660/1/2013_MônicaSampaiodeCarvalho.pdf. Cited 23 Feb 2023.

[37] Ministério da Saúde. Programa Nacional Telessaúde Brasil Redes. Brasília-DF: Ministério da Saúde, 2007. Available from em: https://aps.bvs.br/programa-nacional-telessaude-brasil-redes/. Cited 19 Feb 2023.

[38] Taveira ZZ, Scherer MDA, Diehl EE. Implantação da telessaúde na atenção à saúde indígena no Brasil. Cad Saude Publica. 2014;30(8):1793–7. 10.1590/0102-311X00026214. 10.1590/0102-311x00026214 25210918

[39] GARCIA A.C.P. Gestão do Trabalho e da Educação na Saúde: Uma Reconstrução Histórica e Política. Rio de Janeiro-RJ: Universidade do Estado do Rio de Janeiro, 2010: 173. Available from: https://www.bdtd.uerj.br:8443/bitstream/1/4628/1/Ana%20Claudia%20P%20Garcia-tese.pdf. Cited 19 Feb 2023.

[40] Ministério da Saúde, Ministério da Educação. Portaria Interministerial n. 1.001 de 22 de outubro de 2009 - Institui o Programa Nacional de Apoio à Formação de Médicos Especialistas em Áreas Estratégicas - PRÓ-RESIDÊNCIA. 2009. Brasília-DF: Ministério da Saúde e da Educação. Available from: http://portal.mec.gov.br/index.php?option=com_docman&view=download&alias=1682-port-1001&Itemid=30192. Cited 19 Feb 2023.

[41] Ministério da Saúde. Plano Nacional de Fortalecimento das Residências em Saúde. Brasília-DF: Ministério da Saúde, 2021. Available from: https://www.gov.br/saude/pt-br/composicao/sgtes/publicacoes/publicacao_plano-nacional-de-fortalecimento-das-residencias-em-saude_17-03-2021-versao-web.pdf. Cited 20 Feb 2023.

[42] Kemper ES, Tasca R, Harzheim E, Suárez Jiménez JM, Hadad J, De Sousa MF. Universal health coverage and the more doctors physician recruitment program (Programa Mais Médicos) in Brazil. Rev Panam Salud Publica/Pan Am J Public Heal. 2018;42:1–5. 10.26633/RPSP.2018.1 . 10.26633/RPSP.2018.1 PMC6386144 31093032

[43] Nassar LM, Passador JL, Pereira Júnior GA. Mais Médicos Program: Identification and Analysis of Open Medical Undergraduate Programs. Saude e Soc. 2022;31(4). 10.1590/S0104-12902022200878pt .

[44] Presidência da República, Casa Civil, Subchefia para Assuntos Jurídicos LEI No 10.260, DE 12 DE JULHO DE 2001. Dispõe sobre o Fundo de Financiamento ao estudante do Ensino S. Brasília-DF: Presidência da República, 2001. Available from: http://www.planalto.gov.br/ccivil_03/leis/leis_2001/l10260.htm. Cited 23 Feb 2023.

[45] Presidência da República, Casa Civil, Subchefia para Assuntos Jurídicos. Lei número 11.06, de 13 de janeiro de 2005. Institui o Programa Universidade para Todos - PROUNI, regula a atuação de entidades beneficentes de assistência social no ensino superior; altera a Lei n^o^ 10.891, de 9 de julho de 2004, e dá outras providências [Internet]. Brasília-DF: Presidência da República. 2004:12. Available from: https://www.planalto.gov.br/ccivil_03/_ato2004-2006/2005/lei/l11096.htm. Cited 24 Feb 2023.

[46] Chaves VLJ (2010). Expansão da privatização/mercantilização do ensino superior Brasileiro: a formação dos oligopólios. Educ Soc.

[47] Ministério da Educação. Política Nacional de Expansão das Escolas Médicas das IFES e CAMEM [Internet]. Brasília-DF: Ministério da Educação. 2022:1. Available from: https://www.gov.br/mec/pt-br/acesso-a-informacao/institucional/secretarias/secretaria-de-educacao-superior/escolas-medicas. Cited 24 Feb 2023.

[48] Araújo EMD, Galimbertti PA (2013). A colaboração interprofissional na estratégia saáde da família. Psicol e Soc.

[49] de Figueiredo AM, de Lima KC, Massuda A, de Azevedo GD (2022). Políticas de ampliação do acesso ao ensino superior e mudança no perfil de egressos de medicina no Brasil: um estudo transversal. Cien Saude Colet.

[50] Presidência da República, Casa Civil, Subchefia para Assuntos Jurídicos. Lei número 10.861, de 14 de abril de 2004. Institui o Sistema Nacional de Avaliação da Educação Superior – SINAES e dá outras providências. Brasília-DF: Presidência da República. 2004:5. Available from: https://www.planalto.gov.br/ccivil_03/_ato2004-2006/2004/lei/l10.861.htm. Cited 24 Feb 2023.

[51] Medicina CF de. SAEME-CFM: Entenda o Sistema de Acreditação de Escolas Médicas. Brasília-DF: Conselho Federal de Medicina. 2022:1. Available from: https://crmac.org.br/noticias/saeme-cfm-entenda-o-sistema-de-acreditacao-de-escolas-medicas/. Cited 24 Feb 2023.

